# Substantial Alterations of the Cutaneous Bacterial Biota in Psoriatic Lesions

**DOI:** 10.1371/journal.pone.0002719

**Published:** 2008-07-23

**Authors:** Zhan Gao, Chi-hong Tseng, Bruce E. Strober, Zhiheng Pei, Martin J. Blaser

**Affiliations:** 1 Department of Medicine, New York University School of Medicine, New York, New York, United States of America; 2 Department of Environmental Medicine, New York University School of Medicine, New York, New York, United States of America; 3 Department of Dermatology, New York University School of Medicine, New York, New York, United States of America; 4 Department of Pathology, New York University School of Medicine, New York, New York, United States of America; 5 Department of Microbiology, New York University School of Medicine, New York, New York, United States of America; 6 New York Harbor Veterans Affairs Medical Center, New York, New York, United States of America; Centre for DNA Fingerprinting and Diagnostics, India

## Abstract

For psoriasis, an idiopathic inflammatory disorder of the skin, the microbial biota has not been defined using cultivation-independent methods. We used broad-range 16S rDNA PCR for archaea and bacteria to examine the microbiota of normal and psoriatic skin. From 6 patients, 19 cutaneous samples (13 from diseased skin and 6 from normal skin) were obtained. Extracted DNA was subjected to the broad range PCR, and 1,925 cloned products were compared with 2,038 products previously reported from healthy persons. Using 98% sequence identity as a species boundary, 1,841 (95.6%) clones were similar to known bacterial 16S rDNA, representing 6 phyla, 86 genera, or 189 species-level operational taxonomic unit (SLOTU); 84 (4.4%) clones with <98% identity probably represented novel species. The most abundant and diverse phylum populating the psoriatic lesions was *Firmicutes* (46.2%), significantly (*P*<0.001) overrepresented, compared to the samples from uninvolved skin of the patients (39.0%) and healthy persons (24.4%). In contrast, *Actinobacteria,* the most prevalent and diverse phylum in normal skin samples from both healthy persons (47.6%) and the patients (47.8%), was significantly (*P*<0.01) underrepresented in the psoriatic lesion samples (37.3%). Representation of *Propionibacterium* species were lower in the psoriatic lesions (2.9±5.5%) than from normal persons (21.1±18.2%; *P*<0.001), whereas normal skin from the psoriatic patients showed intermediate levels (12.3±21.6%). We conclude that psoriasis is associated with substantial alteration in the composition and representation of the cutaneous bacterial biota.

## Introduction

The microbiota of humans is vast in extent and in its diversity, but little characterized [1], [2]. The major sites for our indigenous microbiota are the oropharynx, gastrointestinal tract, vagina, and skin [3]–[9]. Historically, the microbiota was defined by the isolation of organisms in culture, but substantial proportions of bacterial and fugal species are fastidious, resisting cultivation [2], [4]. This also is true for human skin, for which recent analyses using molecular methods have revealed a microbiota of considerable diversity, much greater than anticipated from culture-based studies [10]–[12]. In addition to characterizing skin from healthy persons, use of such approaches may be of value in understanding disease conditions.

Psoriasis, a chronic inflammatory condition of the skin, present in about 2% of the world's population [13], also affects cardiovascular disease risk 14, 15. The histopathological features of psoriasis include hyperkeratosis, hyperproliferation of keratinocytes, infiltration of skin by immune cells, and angiogenesis [13]. Although the causes of psoriasis are poorly understood, the disease appears to result from a combination of genetic and environmental factors; putative loci for genetic susceptibility to psoriasis have been reported on the basis of genome-wide linkage studies [13].

An extensive literature also has suggested that bacteria, including *Staphylococcus aureus* [16] and *Streptococcus pyogenes* [17], could play a role in the induction and maintenance of psoriasis [17]–[20], and group A Streptococcus (GAS) antigens or superantigens have been implicated in psoriasis pathogenesis in genetically predisposed individuals [17], [21], [22]. Study of the microbiota of patients with psoriasis is thus important, and complementary to genetic and immunological research [23], [24].

Although most current knowledge of the human skin microbiota derives from cultivation studies [5], [25], culture-independent molecular approaches have created a new basis [10]–[12]. One approach, based on sequencing 16S rRNA genes conserved in all prokaryotes, permits analysis of variable regions that allow identification of particular species, inferences about phylogenetic relationships with known bacteria, and construction of population maps of the indigenous biota [1], [2], [6].

The aim of this study was to analyze the composition of the microbiota in skin from patients with psoriasis to determine whether bacterial populations in psoriatic lesions differ from unaffected skin, and from skin from healthy persons.

## Results

### Species richness and diversity

**Figure 1:**
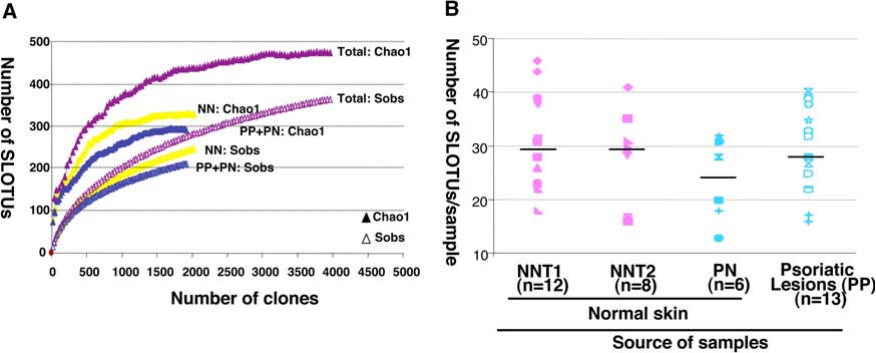
Diversity of bacterial species in 39 skin specimens from 12 subjects. Panel A. Collector's curves of SLOTU richness of pooled skin samples from six healthy subjects and six patients with psoriasis. Each curve reflects the observed (Sobs) or estimated (Chao1) richness values obtained in 3 different groups of 16S rDNA clones. PP+PN indicates the 1,925 (1,314 from psoriatic lesion and 611 from uninvolved skin, respectively), clones from six patients with psoriasis, added to the data set in an arbitrary order. NN includes 2,038 clones from healthy persons reported in a prior study [11]. Overall, the sequences detected from the (Total) 39 human skin specimens from these studies represent 366 different SLOTUs, whereas the Chao1 score estimates that the skin bacterial biota contains 476 SLOTUs. Based on this prediction, Good's estimator of coverage is 96.9%. Panel B. Diversity at the species level. The 20 samples from 6 healthy subjects (pink) and 19 samples from 6 patients with psoriasis (blue) are indicated by the color designations. NNT1 (n = 12) represents left and right forearm skin for six subjects, NNT2 (n = 8) represents the resampling of four subjects 8–10 months later [11]. PN (n = 6) represents uninvolved skin from six patients with psoriasis. The samples from healthy subjects and from uninvolved skin from psoriatic patients are indicated with solid symbols, and the samples from psoriatic lesions are indicated with open symbols. All samples from the same subject are indicated by the same symbol. For each group, the horizontal line indicates the median of the number of SLOTU.

Estimations of species coverage, richness, evenness, and diversity were calculated for the combined data set of 3,963 clones, as well as for subsets of samples defined based on sources of specimens [healthy subjects or subjects with psoriasis] and pathology [psoriatic lesion or uninvolved skin] (Table 1). Good's coverage, which accounts for both diversity and abundance, at 97% for the overall sequence set, indicated that the small subunit rRNA gene (16S rDNA) sequences identified in these samples represent the majority of bacterial sequences present in the human skin samples under study. The Chao1 estimator of total species richness was 476 (Figure 1, Panel A); however, because it did not plateau with the current sequencing effort due to rare species, it likely is a minimal estimate. Ecological diversity also was estimated by Simpson's index of diversity, the Shannon–Weaver index, and evenness, which take into account relative abundances (Table 1). The samples from the lesions of the patients with psoriasis contain the most diverse taxa, with the highest values in all three of these measures.

**Table 1:** Sequence diversity and library coverage estimations

	NNT1^a^ (n = 12)	NNT2^b^ (n = 8)	NN^c^ (n = 20)	PN^d^ (n = 6)	PP^e^ (n = 13)	PN+PP (n = 19)	All (n = 39)
Number of clones	1,221	817	2,038	611	1,314	1,925	3,963
SLOTUs (S)	182	130	247	95	188	212	366
Singletons	67	46	91	38	73	77	125
Simpson's index of diversity (1-D)	0.94	0.93	0.94	0.94	0.97^f^	0.96	0.96
Shannon-Weaver(H)	4.04	3.68	4.13	3.53	4.21^g^	4.18	4.40
Evenness(H/lnS)	0.78	0.76	0.75	0.78	0.80^h^	0.78	0.75
Good's estimator of coverage (%)	94.5	94.4	95.5	93.8	94.4	96.0	96.9
Chao1	246.1	161.1	328.2	135.1	276.8	292.1	476.0
Chao1 standard deviation	20.7	12.0	22.4	17.3	28.0	24.2	25.8

a NNT1: 12 samples from six healthy people, reported in a prior study (11)

b NNT2: Eight samples from four of the six healthy persons collected 8–10 months later (11)

c NN: All 20 samples from the six healthy persons (NNT1+NNT2).

d PN: Six samples from normal skin of six patients with psoriasis.

e PP: 13 samples from psoriatic lesions from six patients with psoriasis.

f P<0.001 compared with NN and PN.

g P<0.001 compared with PN, P = 0.3 compared with NN.

h P<0.001 compared with NN, P = 0.4 compared with PN.

### Phylogenetic analyses

**Figure 2:**
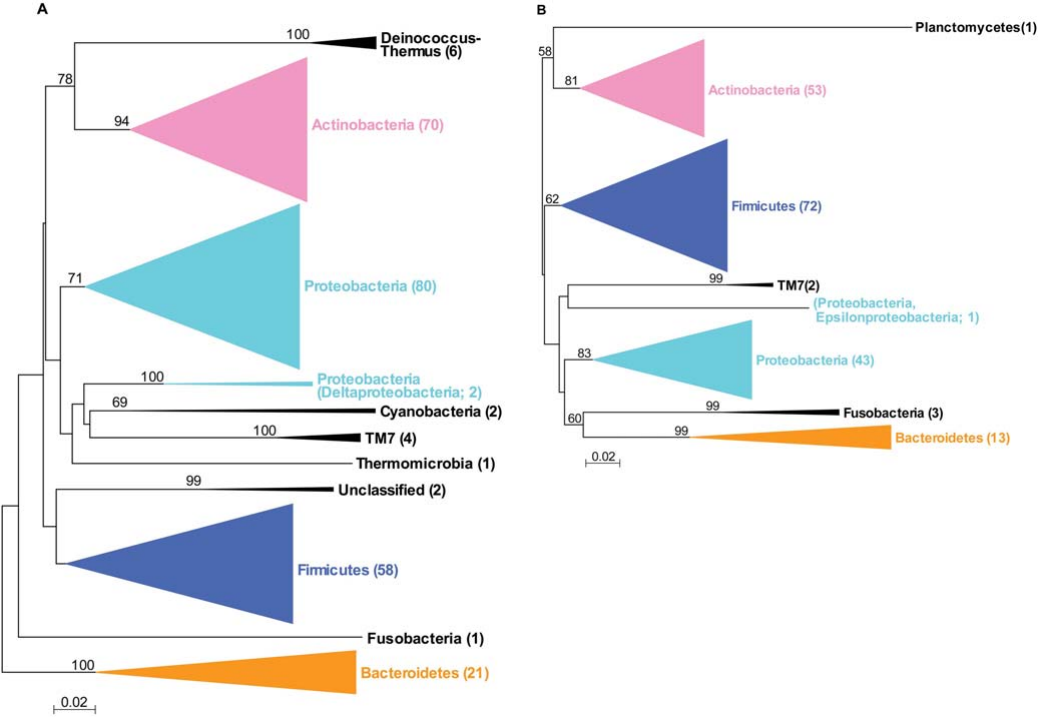
Phylogenetic analysis of bacterial 16S rDNA detected in 39 samples from human skin. From 3,963 clones, sequences representing 11 bacterial phyla and 366 SLOTUs were observed. The numbers in parentheses indicate the number of SLOTUs in each phylum. Alignments were done with Greengenes, and misalignments were manually in MEGA 4.0 [38], evolutionary distances were calculated with the Jukes–Cantor algorithm, and phylogenetic trees were determined by the Neighbor-Joining method; with 1,000 trees generated, bootstrap confidence levels are shown at tree nodes for values ≥50%. Panel A: Phylogenetic analysis of 20 samples from 6 healthy subjects. Panel B: Phylogenetic analysis of 13 samples from the lesions of 6 patients with psoriasis.

The 16S clone libraries from the six patients with psoriasis yielded 1,314 and 611 interpretable unique sequences for the lesions and normal skin samples, respectively. According to the Ribosomal Database Project II (RDP II) database, these were grouped to 8 phyla, 94 genera, and 212 species-level operational taxonomic units (SLOTUs) at 98% identity. In total, 1,841 cloned sequences were similar to those of known bacterial isolates, representing 6 phyla, 86 genera, and 189 SLOTUs. A total of 84 (4.4%) clones were <98% identical to current GenBank entries, and were grouped into 5 phyla, 16 genera, and 23 novel phylotypes. In comparison, for 20 skin samples from 6 healthy subjects, we previously detected 247 SLOTUs, which belonged in 10 phyla [11]. The numbers of species per skin sample was not significantly different between the healthy subjects and those with psoriasis (Figure 1, Panel B). One sample from a patient with psoriasis yielded one sequence representing another phylum, *Planctomycetes*, comprised of aquatic bacteria found in fresh and marine water samples [26]. Overall, the bacteria detected from the 39 human skin specimens from this and the prior study comprise 366 different SLOTUs (Table 2, Figure 2 and Figure S1). Archaea were not identified in any of the 39 samples from healthy persons and patients with psoriasis.

**Table 2:** Number of genera and SLOTU detected in 39 human skin samples

Category	Normal subjects a		Psoriatic subjects		All (n = 39)
	Time 1 (n = 12)	Time 2 (n = 8)	Normal skin (n = 6)	Lesions (n = 13)	
No. of samples	12	8	6	13	39
No. of clones	1,221	817	611	1,314	3,963
Total No. of genera	91	68	43	86	166
Genera/sample^b^	19.0±6.6	17.8±5.5	11.8±4.6	17.8±6.9	17.3±6.6
Total No. of SLOTU	182	130	95	188	366
SLOTU/sample^b^	30.8±9.1	28.5±8.4	23.7±7.8	29.2±7.9	28.7±8.4
Total No. of novel SLOTU	30	19	12	22	66
Novel SLOTU /sample^b^	3.3±3.5	3.4±3.5	2.2±1.5	2.0±1.9	2.6±2.7

a From a prior study (11)

b Mean±SD.

### Distribution at the phylum level

*Firmicutes* and *Actinobacteria,* the dominant phyla in samples of the healthy and diseased skin, were found in each sample, representing >80% of the clones (Table 3). The most abundant and diverse phylum populating the psoriatic lesions was *Firmicutes* (46.2%), significantly (*P*<0.001) overrepresented compared to the uninvolved skin samples of the same patients (39.0%), as well as from healthy persons (24.4%). In contrast, *Actinobacteria,* the most prevalent and diverse phylum in the samples from normal skin of both healthy persons (47.6%) and from the psoriasis patients (47.8%), was significantly (*P*<0.01) lower (37.3%) in the psoriatic lesions. Sequences representing the phylum *Proteobacteria* were detected less frequently from psoriatic lesion samples (11.4%), compared with samples from healthy persons (21.9%, *P*<0.001).

**Table 3:** Phyla detected in skin samples

Phylum	Percent of clones/sample(Mean±SD)				
	Normal subjects a			Psoriatic subjects	
	Time 1 (n = 1,221)^b^	Time 2 (n = 817)	NN (n = 2,038)	(PN) Normal skin (n = 611)	(PP) Lesions (n = 1,314)
*Actinobacteria*	51.6±19.8	41.6±17.2	47.6 ±19.0	47.8±39.2	37.3±26.1 c
*Firmicutes*	23.7±11.2	25.3±19.2	24.4±14.5	39.0±32.8	46.2±21.6 d
*Proteobacteria*	19.3±17.5	25.8±20.0	21.9±18.3	10.1±16.7	11.4±11.5 e
*Bacteroidetes*	1.5±2.4	3.6±3.2	2.4±2.9	2.9±7.2	4.5±7.6
*Cyanobacteria*	0.2±0.6	0.5±1.1	0.3±0.8	0.2±0.4	0
*Fusobacteria*	0	0.1±0.4	0.1±0.2	0	0.2±0.4
*Planctomycetes*	0	0	0	0	0.1±0.3
*TM7*	0	0.7±1.4	0.3±0.9	0	0.2±0.8
*Deinococcus-Thermus*	2.8±6.6	2.2±4.2	2.6±5.6	0	0
*Thermomicrobia*	0.1±0.3	0	0.1±0.2	0	0
Unclassified	0.7±1.7	0.1±0.4	0.5±1.3	0	0

a From a prior study (11)

b Number of clones studied.

c P<0.001 compared with NN, and <0.001 compared with normal skin.

d P<0.001 compared with NN, and 0.003 compared with normal skin.

e P<0.001 compared with NN, and 0.37 compared with normal skin.

### Distribution at the genus level

**Figure 3:**
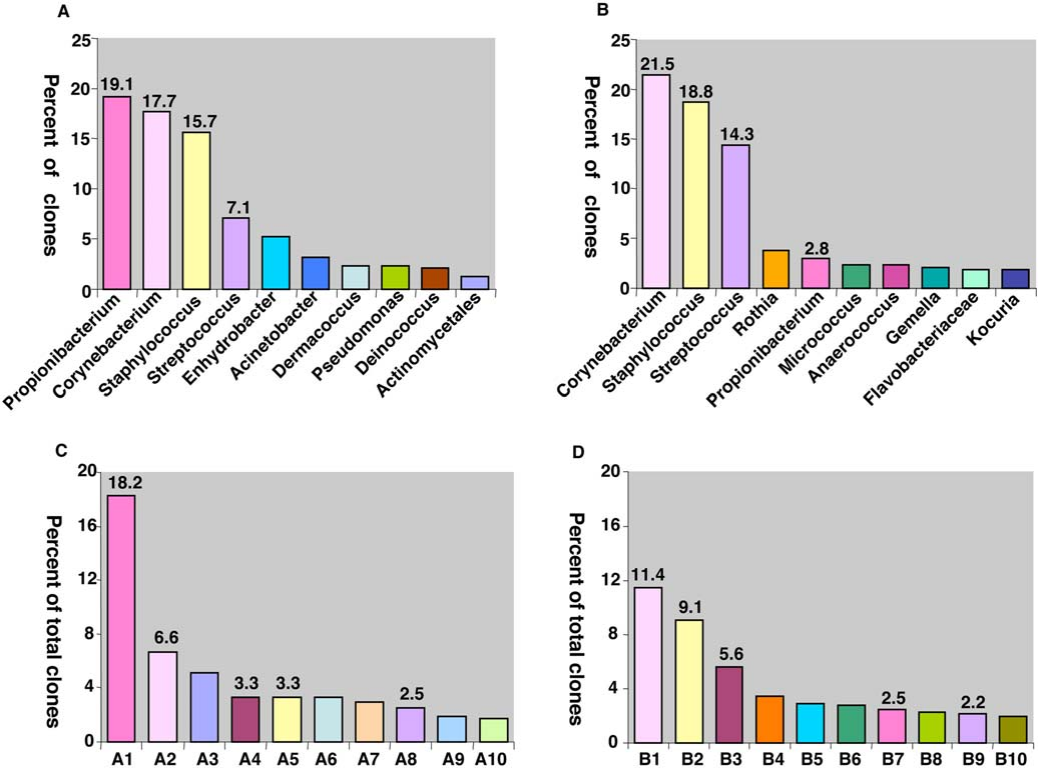
The most common bacterial genera (Panels A, B) and bacterial (Panels C, D) species found in human skin, based on 16S rDNA clones. Panel A. Samples from healthy persons (n = 20) and normal skin (n = 6) of patients with psoriasis (n = 2,649 clones). The ratio of *Streptococcus* to *Propionibacterium* is 0.4. For the four most common genera, the percentage of clones is shown. Panel B. Samples from the lesions (n = 13) of patients with psoriasis (n = 1,314 clones). The ratio of *Streptococcus* to *Propionibacterium* is 5.0 Panel C. Samples from healthy persons (n = 20) and normal skin (n = 6) of patients with psoriasis (n = 2,649 clones). The ratio of *Streptococcus mitis* to *Propionibacterium acnes is* 0.2. Panel D. Samples from the lesions (n = 13) of patients with psoriasis (n = 1,314 clones). The ratio of *Streptococcus mitis* to *Propionibacterium acnes* is 2.5. Code: *Propionibacterium acnes* (A1, B7); *Corynebacterium tuberculostearicum* (A2, B1); *Staphylococcus hominis* (A5, B2); *Streptococcus mitis* (A4, B3); *Staphylococcus epidermidis* (A8, B9); *Enhydrobacter aerosaccus* (A3); *Staphylococcus capitis* (A6); *Staphylococcus caprae* (A7); *Dermacoccus* AF409025 (A9)*; Corynebacterium mucifaciens* (A10); *Corynebacterium simulans* (B4); *Rothia mucilaginosa* (B5); *Staphylococcus aureus* (B6); *Streptococcus salivarius* (B8*); Flavobacteriaceae* DQ337018 (B10).

In total, 166 genera were detected in the 39 samples from human skin, although 10 of the most common genera accounted for the majority of clones from both the healthy and diseased skin samples (Table S1). Only 20 genera were found in all 4 groups of specimens (NNT1, NNT2, PN and PP), but none of the genera was found in every sample. *Corynebacterium, Staphylococcus, Streptococcus,* and *Propionibacterium* were the dominant genera in the samples from normal skin, including healthy persons and patients with psoriasis, as well as from the psoriatic lesions, accounting for >50% of all clones. Clones representing the genus *Streptococcus* were detected significantly more frequently (15.2±10.4%) from psoriatic lesion samples (*P*<0.001) than from the uninvolved skin samples of the patients (3.4±2.5%). In contrast, *Propionibacterium* species represented 21.1±18.2% of the total clones in the samples from the healthy subjects and in the normal skin from psoriatic patients (12.3±21.6%), significantly higher than in the psoriatic lesions (2.9±5.5%) (*P*<0.001). The most common genera detected in the samples from normal and diseased skin were similar (Figure 3), but the proportions differed (Panels A and B). The ratio of *Streptococcus* to *Propionibacterium* (S/P ratio) in the 2,649 clones from healthy persons and normal skin of patients with psoriasis was 0.4. In contrast, the S/P ratio (5.0) in the 1,314 clones from psoriatic lesion samples was significantly (*P* = 0.01) higher.

### Distribution at the SLOTU level

Table S2 lists the 366 SLOTUs represented in superficial human skin, reflecting the prior [11] and the current study. The most numerous species identified belonged to the genera *Corynebacterium, Staphylococcus, Streptococcus, and Anaerococcus*. In total, 32 SLOTUs (8.7%) belonging to 15 genera were detected in all four groups of specimens, accounting for 63.2% of all clones. The four most prevalent bacterial species in each of the different skin specimen groups, accounted for 29.6∼44.0 % of all clones in that group (Table S3). *Propionibacterium acnes* was the most prevalent species in the samples from the healthy subjects (Mean±SD:20.2±16.0%) and from the unaffected skin of the patients with psoriasis (Figure 3, Panel C). Representation of *P. acnes* was much lower (2.6±5.1%) in the samples from the lesions of the patients with psoriasis than in the samples from normal persons (Figure 3, Panel D) (*P*<0.001); normal skin from psoriasis patients showed intermediate levels (11.7±20.4%). *Staphylococcus aureus,* long regarded as being associated with psoriasis [16] only represented 1.1% and 2.8% of the clones from the unaffected and diseased samples of the patients, respectively (Table S2). The distribution of gram-positive and gram-negative anaerobic and facultative bacterial species in psoriatic lesions in the six patients differed from their own normal skin (Table S4). The major differences were found in anaerobic bacterial species between the two groups (*P*<0.05), with the most significant difference found for *Propionibacterium* species (*P*<0.001).

### Double principal coordinate analysis (DPCoA) of the samples from human skin

**Figure 4:**
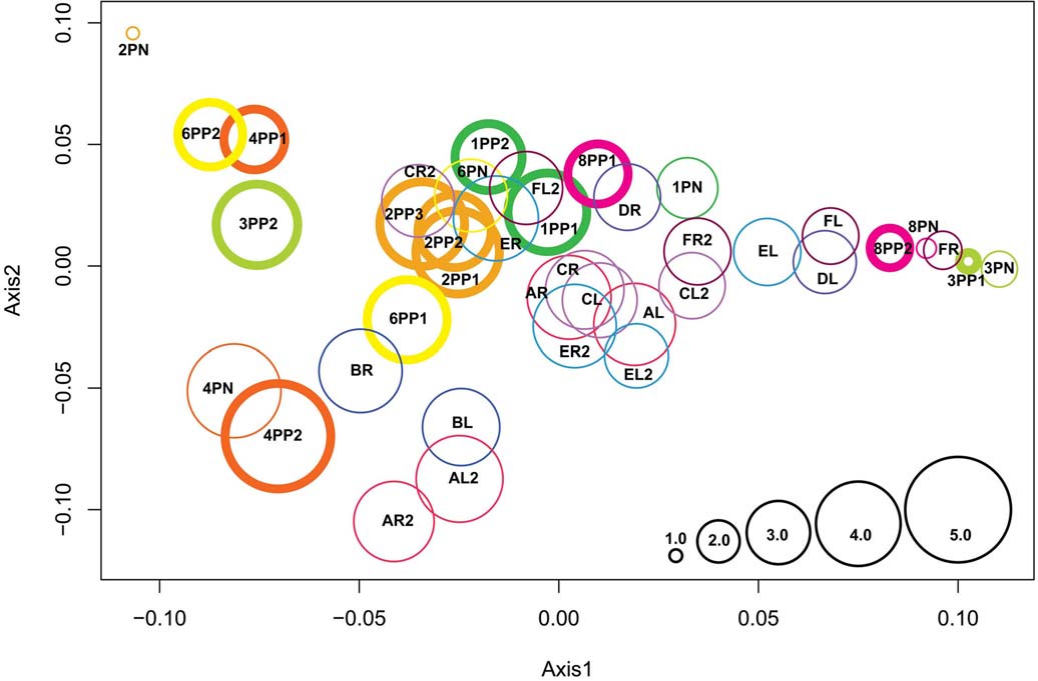
DPCoA of SLOTU relatedness in 39 human skin samples obtained from healthy subjects and psoriatic patients. The healthy subjects were designated A–F, and at each sampling both left (L) and right (R) forearm skin was examined. In four subjects, new specimens were obtained 8–10 months later (e.g. designated AL2). The psoriatic patients were designated 1, 2, 3, 4, 6, and 8, PN indicates that sample is from uninvolved skin and PP is from psoriatic lesions. In a representation of the first two orthogonal principal axes, based on a sample dissimilarity matrix, samples from the same subjects at the same time point are plotted by using the same color, and the normal skin and psoriatic lesion samples are indicated by thin and thick circle walls, respectively. The size of each circle is proportional to the sample's Rao diversity index. The scale (bottom right) indicates the relative diversity of the populations within the circles, with the test sample of smallest diversity (2PN) indexed as 1.0.

Similarities in SLOTU distributions between skin samples were evaluated using DPCoA (Figure 4). Four hypotheses concerning the overall grouping of samples were tested. First, analysis using all 39 samples of human skin from 12 persons (6 healthy persons and 6 patients with psoriasis) showed that those samples from the same subject were more similar to each other than to samples from other subjects (*P*<0.001). The same result was found when only analyzing the newly obtained 19 samples from the six patients with psoriasis (*P* =  0.006). Second, in analysis of the 19 samples from the patients with psoriasis, those obtained from psoriatic lesions were not significantly different than those from unaffected skin from the same patient, although these was an overall trend (*P* = 0.062). Third, the samples of psoriatic skin from the patients (n = 13) were clustered together, compared to samples of normal skin from healthy subjects (n = 20) (*P* = 0.001). Fourth, the samples obtained from unaffected skin from the patients with psoriasis (n = 6) were not significantly different from those from normal skin of healthy subjects (n = 20) (*P* = 0.12).

The 16S rDNA clone library profiles from all 39 human skin samples (Figure S1) also were compared using Unifrac analysis (Figure S2); samples from the same person also often clustered together, as did clinical group of specimens.

## Discussion

The skin is a complex habitat with many residential bacterial species. A sterile milieu prenatally, human skin becomes host to resident bacteria soon after birth [27]. The normal microbiota acts as a barrier against colonization of potentially pathogenic introduced microbes and against overgrowth of already present opportunistic microorganisms. Microbial metabolism also potentially has both beneficial and harmful effects for the host [27], [28]. However, the host-interactions of human skin microbiota remain largely unexplored.

We developed approaches to the assessment of the major microbial populations in the human skin using molecular techniques, based on ribosomal operons and PCR [11], [12], and we now apply this technique to understanding the microbiology of skin involved with psoriasis. By adding more subjects and skin locations to our studies, the number of bacterial species continued to expand, with nearly 500 SLOTUs projected, and no plateau of the collectors curve. These observations suggest that human skin is a highly diverse ecosystem, with levels of bacterial diversity perhaps approaching that of the colon [4].

We sought to determine whether the involved skin from subjects with psoriasis differed in colonization patterns compared with their own uninvolved skin, and with skin from healthy persons. Although there were general similarities in the types of bacteria present and in general proportions, important differences emerged. First, the overall ecological diversity of the microbial population overlaying the lesions was greater than for other skin samples (Table 1). This may reflect the heterogeneity of the types of lesions sampled, compared with the relatively homogeneous uninvolved skin. Second, there were significant differences in the distribution of *Actinobacteria, Proteobacteria,* and *Firmicutes*, the three major phyla populating normal skin, with the former two phyla underrepresented, and the latter overrepresented, as well as significant differences for minor phylum *Bacteroidetes*. Although these represent the aggregation of numerous genera and species, the significant differences suggest that there has been substantial ecological disturbance, in contrast to the observations for fungal species in relation to psoriasis [12]. Third, there was a striking and progressive underrepresentation of the genus *Propionibacterium*, and its major human species *P. acnes,* as the samples from healthy persons, healthy skin from psoriasis patients, and psoriatic lesions were compared (Table S3, Figure 3). The use of the same PCR primers for all groups of specimens suggest that differences in amplification efficiencies that occur when different primer pairs are used can not explain the observations.

*Propionibacterium*, and *P. acnes* in particular, are dominant organisms in normal skin [29], as shown in culture-based studies [10], and in our molecular analyses [11]. A diminution in these numbers could be a reflection of the disordered ecological niches that are inhospitable to these organisms, could play a role in the pathogenesis of the disease, or could represent the effects of various treatments of psoriasis. The lack of any treatment of the subjects in this study, and that their unaffected skin showed an intermediate diminution provides evidence against any effect of treatments, but suggests that ecological disordering could be occurring, although at a subclinical level. Alternatively, the presence *P. acnes* has some protective role. One possible explanation is that *P. acnes* has immunomodulatory constituents [30] that could be playing a role in signaling human cells [31]. Lastly, *P. acnes* may be displaced by more aggressive organisms or conversely, might protect the skin against more aggressive microbes, and its diminution reduces that barrier. In one scenario, *P. acnes* levels are affected by other phenomena; in the other, it plays a causative role. Favoring these microbial competition hypotheses is the greater representation of streptococcal and staphylococcal species in the psoriatic lesions, however, Group A *Streptococcus pyogenes* and *Staphylococcus aureus* were absent or minimally present.

Future studies must involve more quantitative techniques to determine whether the relative representations that we observe reflect absolute differences or not. Clearly, examination of greater numbers of lesions, with different clinical and pathological phenotypes, will provide more accurate stratification of the microbial associations. Even if the diminished representation of *Propionibacterium* is merely a secondary marker of the psoriatic process, understanding its presence could aid in the diagnosis and assessment of the efficiency of therapies. This study did not explore differences at the *P. acnes* strain level, but such also could be relevant in future examinations.

Finally, this work raises the possibility that knowledge of specific components of the microbiota in disease could lead to attempts to reduce numbers of over-represented organisms, and to increase populations of commonly occurring resident microbes that are diminished in disease. Such a probiotic (or a prebiotic) approach will require much greater knowledge of the specific characteristics of key human microbial constituents, and determinations of microbial genotypes and phenotypes in relation to disease.

## Materials and Methods

### Clinical specimens

This study was approved by the NYU Institutional Review Board and all subjects provided written informed consent. Six patients with psoriasis were analyzed. From each patient, at least 3 skin samples, including one sample from unaffected skin and at least two samples from psoriatic lesions, were studied. Subjects included three men and three women; details of their demographic and clinical characteristics are shown in Table S5. None of the patients were currently receiving therapy, either topical, ultraviolet, or systemic, for psoriasis.

### Specimen processing

Methods for sampling superficial skin have been described [11]. Lesions differing in the extent of erythema, swelling, and scaling, as well as normal skin were sampled by swabbing the skin with a cotton pledget that had been soaked in sterile 0.15M NaCl with 0.1% Tween-20 (Fisher Scientific, Fair Lawn, NJ). DNA was extracted from the swabs in a PCR-free clean-room by using the DNeasy Tissue Kit (Qiagen, Chatsworth, CA); since Gram-positive bacteria are more resistant to lysis than Gram-negative organisms, the manufacturer's protocol for genomic DNA isolation from Gram-positive bacteria was followed. Samples were eluted in 100 µl AE buffer, and to eliminate bacterial or DNA contamination, the enzymatic lysis buffer was passed through a micro-centrifuge filter (MW threshold 30,000 Daltons, Amicon, Bedford, MA) at 747×g for 20 min.

### 16S rDNA PCR amplification

Two different primer sets were used for PCR: one set (8F: 5′-AGA GTT TGA TYM TGG CTC AG and 1510R: 5′-TAC GGY TAC CTT GTT ACG ACT T) was specific to the 16S rRNA gene of bacteria, which amplifies ∼1.5 kb PCR products, based on positions 8 to 1513 of the *E. coli* 16S rRNA gene [8], [32], [33]. To each 5 µl of the suspension of extracted template DNA was added 45 µl of a PCR mixture containing 5 µl of 10x PCR buffer (Qiagen), 2.5 mM MgCl_2_, 200 µM each dNTP, 20pmol of each primer, and 1.25units of TaqDNA polymerase. PCR was performed for 2 min at 94°C, followed by 30 amplification cycles of 45 s at 94°C, 30 s at 52°C, and 90 s at 72°C, with a final cycle for 20 min at 72°C. The second primer set [Arch21F: 5′-TTC CGG TTG ATC CYG CCG GA-3′ [34], and A19R: 5′-YCC GGC GTT GAM TCC AAT T-3′ [7]] was specific to archaea. PCR was carried out in a 50 µl reaction containing 1×PCR buffer, 2 mM MgCl_2_, 200 µM each dNTP, 10pmol of each primer, 1.25units of TaqDNA polymerase and 5 µl of DNA template. The amplification procedure comprised of one cycle for 4 min at 95°C followed by 35 cycles of 30 s at 95°C, 1 min at 55°C and 1 min at 72°C, and one cycle of 5 min at 72°C. The results of PCR amplifications were examined by electrophoresis on 1% agarose gels.

### 16S rDNA clone libraries

The PCR products were separated from free PCR primers using a PCR purification kit (Qiagen), ligated with the pGEM-T-Easy vector (Promega, Madison, WI), and used to transform *E. coli* DH5α competent cells. Putatively positive clones were screened by PCR with Sp6/T7 primers. The cloned inserts underwent sequence analysis using PCR primers 8F and 27R (reverse primer 5′-CGA CAI CCA TGC AIC ACC T, corresponding to position 8 to 1064 of the *E. coli* 16S rRNA). Each sequence was manually edited in conjunction with its chromatogram with Sequencher, adjusting for quality. DNA sequences of approximately 980 bases were obtained initially to determine either identity or approximate phylogenetic position. For those clones containing inserts of ambiguous phylogenetic status, nearly full-length 16S bacterial rRNA sequences (∼1,500 bp) were obtained, using the additional primer, 1510R. For identification of closest relatives, these newly determined sequences were compared to those available in the RDP II (release 9.39) [35] and GenBank (www.ncbi.nlm.gov) databases, using the standard nucleotide-nucleotide BLAST program to ascertain their closest relatives. A novel phylotype was defined as a sequence that differed from the closest GenBank entry by >2%. Data for the novel sequences were submitted to GenBank (GenBank accession numbers: EF419345-EF419425).

### Phylogenetic analyses

DNA sequences of 940 to 1450 bp were obtained for about 100 clones from each sample, for a total of 1,933 clones. All sequences were examined for chimerism by using Chimera Detection at RDP II (release 8.1) and Bellerophon [36]. In total, 8 clones were removed from the phylogenetic analysis. The remaining 1,925 sequences were compared with those of RDP II (release 9.39) [35] and in GenBank to identify SLOTUs (species-level operational taxonomic units), as reported [8]. The sequences were aligned with NAST at Greengenes (http://greengenes.lbl.gov/cgi-bin/nph-index.cgi) [37] Misalignments were manually curated in MEGA 4.0 [38], and then hypervariable regions were masked by using MASK COLUMNS at Greengenes [37]. The phylogenetic trees were generated using MEGA 4.0 [38]. Evolutionary distances were calculated with the Jukes-Cantor algorithm [39]. The statistical strength of the Neighbor-Joining method was assessed by bootstrap resampling (1,000 replicates) [40].

### Statistical analyses

Double Principal Coordinate Analysis (DPCoA) [41] was used to evaluate sample diversity and the relationships amongst samples. This method uses phylotype differences to derive the dissimilarity matrix of samples and to calculate the sample diversity. In this analysis, the dissimilarities between different phylotypes are calculated based on the sum of distances to the common ancestor of two phylotypes on the phylotype tree. To facilitate the visualization of sample dissimilarity and diversity, the first two orthogonal principal axes were obtained based on the sample dissimilarity, and were plotted to show the distribution of samples in a two-dimensional space. The diversity information can be decomposed into within- and between- sample diversity values. This allowed the use of a “pseudo F” [4] statistic (the ratio of within-cluster diversity and between-cluster diversity) to examine possible clustering phenomena, and significance was evaluated by permutation tests. Results from 2,038 sequences from healthy persons, as reported [11], were included for comparison of the microbiota between unaffected skin and psoriatic lesions. UniFrac [42] also was used to assess for significant differences between samples.

## Supporting Information

Table S1 The 10 most common genera found in human skin
(0.05 MB DOC)

Table S2 Representation of 366 bacterial SLOTUs in the human skin
(0.84 MB DOC)

Table S3 The four most common species found in different groups of skin specimens
(0.04 MB DOC)

Table S4 Supplemental information
(0.03 MB DOC)

Table S5 Origin of skin samples
(0.10 MB DOC)

Figure S1 Phylogenetic analysis of bacterial 16S rDNA detected in 39 samples from human skin of 12 persons. From 3,963 clones, sequences representing 11 bacterial phyla and 366 SLOTUs were observed. The numbers in parentheses indicate the number of SLOTUs in each phylum. Alignments were done with Greengenes, and misalignments were manually in MEGA 4.0 (38), evolutionary distances were calculated with the Jukes-Cantor algorithm, and phylogenetic trees were determined by the Neighbor-Joining method; with 1,000 trees generated, bootstrap confidence levels are shown at tree nodes for values ≥50%.
(0.78 MB TIF)

Figure S2 Hierarchical clustering of 39 human skin samples from healthy and psoriasis subjects using weighted Unifrac. The numerical support for nodes present in ≥50% of sequence jackknifing is indicated, based on 1,000 permutations. The healthy subjects were designated A–F, and at each sampling both left (L) and right (R) forearm skin was examined. In four subjects, new specimens were obtained 8–10 months later (e.g. designated AL2). The psoriatic patients were designated 1, 2, 3, 4, 6, and 8, PN indicates that sample is from uninvolved skin and PP is from psoriatic lesions. The highly significant clustering observed relates to samples from the same person.
(1.11 MB TIF)
