# Supplementary material for: Substantial Alterations of the Cutaneous Bacterial Biota in Psoriatic Lesions
Source: PLoS One. 2008 Jul 23;3(7):e2719. doi: 10.1371/journal.pone.0002719 (PMC2447873; doi:10.1371/journal.pone.0002719)
Supplement: Table S1 — The 10 most common genera found in human skin (0.05 MB DOC) [file pone.0002719.s001.doc]

**Table S1. The 10 most common genera found in human skin**

| **Overall**  **rank** | **Genus** | **Mean % of clones ± SD ^a^** | | | | | **Number of subjects** | | | |
| --- | --- | --- | --- | --- | --- | --- | --- | --- | --- | --- |
|  |  | **NNT1 ^b^** | **NNT2 ^c^** | **NN^f^** | **PN ^d^** | **PP ^e^** | **NNT1** | **NNT2** | **PN** | **PP** |
| 1 | *Corynebacterium* | 19.1±15.2 | 7.2±8.1 | 14.3±13.9 | 29.2±30.3 | 22.7±23.6 | 6 | 4 | 6 | 6 |
| 2 | *Staphylococcus* | 11.1±8.3 | 10.5±13.3 | 10.9±10.3 | 32.0±32.1 | 18.4±12.6 | 6 | 4 | 5 | 6 |
| 3 | *Propionibacterium* | 22.1±19.2 | 19.7±17.8 | 21.1±18.2 | 12.3±21.6 | 2.9±5.5 | 6 | 4 | 4 | 4 |
| 4 | *Streptococcus* | 5.8±5.4 | 11.5±14.2 | 8.1±10.0 | 3.4±2.5 | 15.2±10.4 | 6 | 4 | 5 | 6 |
| 5 | *Enhydrobacter* | 2.8±4.4 | 12.5±15.2 | 6.7±11.0 | 0.2±0.4 | 0.7±0.8 | 3 | 2 | 1 | 4 |
| 6 | *Acinetobacter* | 3.7±4.0 | 3.8±4.3 | 3.4±4.0 | 1.0±2.4 | 1.7±3.1 | 6 | 3 | 1 | 3 |
| 7 | *Dermacoccus* | 0.8±1.7 | 6.3±12.2 | 3.3±8.0 | 0 | 1.1±2.3 | 2 | 2 | 0 | 2 |
| 8 | *Pseudomonas* | 2.7±3.9 | 1.0±1.1 | 2.2±3.8 | 2.5±5.5 | 1.4±1.2 | 4 | 4 | 2 | 4 |
| 9 | *Rothia* | 1.8±2.8 | 0.4±0.7 | 1.2±2.3 | 0.5±0.8 | 3.8±3.2 | 5 | 2 | 2 | 5 |
| 10 | *Micrococcus* | 0.5±1.4 | 2.7±4.9 | 1.4±3.3 | 0.2±0.4 | 1.9±2.5 | 2 | 2 | 1 | 5 |
| **Top 10 genera** | | **70.4** | **75.6** | **72.9** | **81.3** | **69.8** |  |  |  |  |

^a^Percent of mean levels in each sample arranged in order of decreasing frequency

^b^NNT1: 12 samples from six healthy people, reported in a prior study (11).

^c^NNT2: Eight samples from four of six healthy people 8-10 months later (11).

^d^PN: Six samples from normal skin from the six patients with psoriasis.

^e^PP: 13 samples from psoriatic lesions from the six patients with psoriasis.

^f^NN **:** NNT1+NNT2
