# Supplementary material for: Substantial Alterations of the Cutaneous Bacterial Biota in Psoriatic Lesions
Source: PLoS One. 2008 Jul 23;3(7):e2719. doi: 10.1371/journal.pone.0002719 (PMC2447873; doi:10.1371/journal.pone.0002719)
Supplement: Table S2 — Representation of 366 bacterial SLOTUs in the human skin (0.84 MB DOC) [file pone.0002719.s002.doc]

**Table S2. Representation of 366 bacterial SLOTUs in the human skin**

| **Phylum** | **Species** | **Number of clones** | | | | |
| --- | --- | --- | --- | --- | --- | --- |
|  |  | **Normal subjects** | | **Subjects with psoriasis** | | **Total** |
|  |  | **NNT1^a^** | **NNT2^b^** | **PN^c^** | **PP^d^** |  |
| **Actinobacteria**  **(n=100)** | *Actinobaculum* AJ298658 93% |  |  |  | 1 | 1 |
|  | *Actinomyces* AF287749 |  |  | 1 |  | 1 |
|  | *Actinomyces* AF385553 | 1 |  | 3 |  | 4 |
|  | *Actinomyces* AJ635359 |  |  |  | 1 | 1 |
|  | *Actinomyces* AY349365 |  |  | 1 |  | 1 |
|  | *Actinomyces naeslundii* | 4 |  |  | 2 | 6 |
|  | *Actinomyces neuii* | 7 | 1 |  | 2 | 10 |
|  | *Actinomyces odontolyticus* |  | 1 |  | 2 | 3 |
|  | *Actinomycetales* AY770698 | 19 | 6 | 8 | 2 | 35 |
|  | *Arsenicicoccus bolidensis* |  | 1 |  |  | 1 |
|  | *Atopobium* AY959044 |  |  |  | 1 | 1 |
|  | *Atopobium vaginae* | 3 |  |  |  | 3 |
|  | *Bifidobacterium* AF275882 |  |  | 1 |  | 1 |
|  | *Bifidobacterium longum biovar Longum* |  |  |  | 1 | 1 |
|  | *Blastococcus* AY234675 91% | 2 |  |  |  | 2 |
|  | *Brevibacterium* X93594 94% |  |  | 1 | 1 | 2 |
|  | *Brevibacterium casei* |  |  |  | 1 | 1 |
|  | *Brevibacterium paucivorans* | 3 |  | 1 | 2 | 6 |
|  | *Candidatus Nostocoida limicola* | 1 |  |  |  | 1 |
|  | *Corynebacterium* AF227828 |  |  | 2 | 3 | 5 |
|  | *Corynebacterium* AF537601 96% | 2 |  |  |  | 2 |
|  | *Corynebacterium* X84253 | 1 |  |  |  | 1 |
|  | *Corynebacterium accolens* | 2 | 4 |  | 6 | 12 |
|  | *Corynebacterium* AF115937 |  |  | 2 |  | 2 |
|  | *Corynebacterium* AF227854 |  |  | 5 | 3 | 8 |
|  | *Corynebacterium* AF543288 | 1 |  |  |  | 1 |
|  | *Corynebacterium afermentans* | 4 | 3 | 1 | 7 | 15 |
|  | *Corynebacterium* AJ438050 95% |  |  | 1 | 4 | 5 |
|  | *Corynebacterium* AJ439345 95% | 1 |  |  |  | 1 |
|  | *Corynebacterium amycolatum* | 23 |  | 16 | 22 | 61 |
|  | *Corynebacterium appendicis* | 7 | 3 | 2 | 5 | 17 |
|  | *Corynebacterium aurimucosum* | 3 |  | 4 | 3 | 10 |
|  | *Corynebacterium* AY244774 94% |  |  |  | 1 | 1 |
|  | *Corynebacterium* AY581888 | 2 |  |  |  | 2 |
|  | *Corynebacterium* AY880057 |  |  |  | 1 | 1 |
|  | *Corynebacterium coyleae* | 2 |  |  |  | 2 |
|  | *Corynebacterium durum* | 3 | 2 |  | 1 | 6 |
|  | *Corynebacterium glaucum* | 1 |  |  |  | 1 |
|  | *Corynebacterium glucuronolyticum* | 6 |  |  |  | 6 |
|  | *Corynebacterium imitans* | 9 |  |  |  | 9 |
|  | *Corynebacterium jeikeium* | 14 | 2 | 16 | 12 | 44 |
|  | *Corynebacterium kroppenstedtii* | 10 | 7 | 2 | 3 | 22 |
|  | *Corynebacterium lipophiloflavum* | 2 |  |  |  | 2 |
|  | *Corynebacterium macginleyi* |  |  | 1 |  | 1 |
|  | *Corynebacterium matruchotii* | 2 |  |  |  | 2 |
|  | *Corynebacterium minutissimum* | 6 |  | 6 | 4 | 16 |
|  | *Corynebacterium mucifaciens* | 29 | 3 | 14 | 9 | 55 |
|  | *Corynebacterium nigricans* | 6 | 2 | 2 |  | 10 |
|  | *Corynebacterium pseudodiphthericum* | 1 |  |  |  | 1 |
|  | *Corynebacterium simulans* | 8 | 2 | 27 | 45 | 82 |
|  | *Corynebacterium singulare* | 5 | 2 | 2 | 3 | 12 |
|  | *Corynebacterium sundsvallense* | 3 |  |  |  | 3 |
|  | *Corynebacterium thomssenii* |  | 2 |  |  | 2 |
|  | *Corynebacterium tuberculostearicum* | 78 | 27 | 71 | 150 | 326 |
|  | *Corynebacterium urealyticum* |  |  | 3 |  | 3 |
|  | *Corynebacterium* X81872 | 1 |  |  | 1 | 2 |
|  | *Corynebacterium* Y10077 96% |  |  | 1 | 1 | 2 |
|  | *Dermabacter hominis* |  |  | 6 | 10 | 16 |
|  | *Dermacoccus* AF409025 95% | 9 | 4 |  |  | 13 |
|  | *Gardnerella vaginalis* | 3 | 3 |  |  | 6 |
|  | *Gordonia bronchialis* | 4 |  |  |  | 4 |
|  | *Gordonia sputi* | 4 | 2 |  |  | 6 |
|  | *Gordonia terrae* | 3 | 1 |  |  | 4 |
|  | *Janibacter melonis* | 1 |  |  |  | 1 |
|  | *Kocuria carniphila* |  |  |  | 2 | 2 |
|  | *Kocuria kristinae* |  |  | 2 | 4 | 6 |
|  | *Kocuria marina* | 1 | 4 |  | 4 | 9 |
|  | *Kocuria palustris* | 2 |  |  | 1 | 3 |
|  | *Kocuria rhizophila* | 8 | 1 |  | 13 | 22 |
|  | *Microbacterium lacticum* |  | 1 |  |  | 1 |
|  | *Dermacoccus* AF409025 | 1 | 47 |  | 19 | 67 |
|  | *Micrococcus* DQ513327 97% |  |  |  | 6 | 6 |
|  | *Micrococcus luteus* | 6 | 22 | 1 | 23 | 52 |
|  | *Microlunatus phosphovorus* | 1 |  |  |  | 1 |
|  | *Mobiluncus curtisii subsp. holmesii* | 1 |  |  |  | 1 |
|  | *Mycobacterium chlorophenolicum* | 1 |  |  |  | 1 |
|  | *Mycobacterium obuense* | 2 |  |  |  | 2 |
|  | *Nakamurella multipartita* | 2 |  |  |  | 2 |
|  | *Propionibacteriaceae* AF285117 |  |  | 4 |  | 4 |
|  | *Propionibacterium* AB042290 |  | 1 |  |  | 1 |
|  | *Propionibacterium* AB108480 |  |  |  | 1 | 1 |
|  | *Propionibacterium acnes* | 252 | 159 | 72 | 33 | 516 |
|  | *Propionibacterium granulosum* | 10 | 1 | 1 | 1 | 13 |
|  | *Propionibacterium* Y17821 | 6 |  | 3 | 2 | 11 |
|  | *Rhodococcus corynebacterioides* | 7 | 3 |  |  | 10 |
|  | *Rhodococcus erythropolis* | 5 | 9 | 1 | 20 | 35 |
|  | *Rothia aeria* | 1 |  |  | 2 | 3 |
|  | *Rothia amarae* |  |  | 2 | 3 | 5 |
|  | *Rothia dentocariosa* | 1 | 1 |  | 4 | 6 |
|  | *Rothia mucilaginosa* | 18 | 2 | 1 | 39 | 60 |
|  | *Rothia nasimurium* | 2 |  |  |  | 2 |
|  | *Rubrobacter xylanophilus* |  |  |  | 1 | 1 |
|  | *Tetrasphaera elongata* | 1 |  |  |  | 1 |
|  | *Tsukamurella pulmonis* |  | 10 |  |  | 10 |
|  | *Tsukamurella tyrosinosolvens* | 3 | 1 |  |  | 4 |
|  | *Turicella otitidis* |  |  | 4 | 1 | 5 |
|  | *Varibaculum cambriense* |  |  |  | 1 | 1 |
|  | *Zimmermannella* AB012589 97% |  |  | 1 | 1 | 2 |
|  | *Zimmermannella alba* |  |  | 1 |  | 1 |
|  | *Zimmermannella* AJ251780 | 1 |  |  |  | 1 |
|  | **Subtotal** | **628** | **340** | **293** | **491** | **1752** |
| **Bacteroidetes**  **(n=29)** | *Bacteroidales* AF385513 | 2 |  |  |  | 2 |
|  | *Bacteroidales* AY349402 |  |  |  | 3 | 3 |
|  | *Bacteroides vulgatus* |  |  |  | 4 | 4 |
|  | *Capnocytophaga* AF385569 |  |  |  | 1 | 1 |
|  | *Chitinophaga* AJ318177 88% | 1 |  |  |  | 1 |
|  | *Chryseobacterium* AJ619057 96% |  | 2 |  |  | 2 |
|  | *Chryseobacterium* DQ314741 |  | 5 |  |  | 5 |
|  | *Chryseobacterium meningosepticum* |  |  |  | 1 | 1 |
|  | *Flavobacteriaceae* AY958777 93% |  |  |  | 2 | 2 |
|  | *Flavobacteriaceae* DQ337018 |  | 10 | 7 | 25 | 42 |
|  | *Flavobacteriaceae* AF502204 | 1 |  |  |  | 1 |
|  | *Flavobacteriaceae* DQ256356 95% |  | 2 | 11 | 15 | 28 |
|  | *Flexibacteraceae* AY279982 87% | 1 |  |  |  | 1 |
|  | *Flexibacteraceae* AM000023 85% |  | 7 |  |  | 7 |
|  | *Flexibacteraceae* AM000023 91% |  | 1 |  |  | 1 |
|  | *Flexibacteraceae* AM000023 89% |  | 2 |  |  | 2 |
|  | *Hymenobacter* D84607 93% | 2 |  |  |  | 2 |
|  | *Porphyromonas* L16493 86% | 1 |  |  |  | 1 |
|  | *Porphyromonas* AY008310 |  |  |  | 4 | 4 |
|  | *Porphyromonas* AY008313 | 1 |  |  |  | 1 |
|  | *Porphyromonas uenonis* |  |  |  | 1 | 1 |
|  | *Prevotella* AY207061 | 1 |  |  |  | 1 |
|  | *Prevotella* AY323525 | 1 |  |  |  | 1 |
|  | *Prevotella* AY880052 |  |  |  | 1 | 1 |
|  | *Prevotella bivia* | 1 |  |  |  | 1 |
|  | *Prevotella corporis* | 1 |  |  | 1 | 2 |
|  | *Prevotella disiens* | 2 |  |  | 1 | 3 |
|  | *Prevotella* L16476 93% | 1 |  |  |  | 1 |
|  | *Prevotella melaninogenica* | 3 |  |  | 1 | 4 |
|  | **Subtotal** | **19** | **29** | **18** | **60** | **126** |
| **Cyanobacteria**  **(n=3)** | *Cyanobacteria* AJ538357 94% | 2 | 1 |  |  | 3 |
|  | *Cyanobacteria* AY853673 83% |  | 3 |  |  | 3 |
|  | *Cyanobacteria* DQ330752 96% |  |  | 1 |  | 1 |
|  | **Subtotal** | **2** | **4** | **1** |  | **7** |
| **Deinococcus**  **(n=6)** | *Deinococcus* AE002076 91% | 2 | 2 |  |  | 4 |
|  | *Deinococcus*  AJ549111 91% | 29 | 2 |  |  | 31 |
|  | *Deinococcus* Y11329 90% | 4 | 8 |  |  | 12 |
|  | *Deinococcus* AY743261 93% |  | 2 |  |  | 2 |
|  | *Deinococcus grandis* |  | 2 |  |  | 2 |
|  | *Deinococcus radiodurans* |  | 2 |  |  | 2 |
|  | **Subtotal** | **35** | **18** |  |  | **53** |
| **Firmicutes**  **(n=103)** | *Abiotrophia defectiva* |  | 2 |  | 1 | 3 |
|  | *Acidaminococcaceae* DQ057390 |  |  |  | 2 | 2 |
|  | *Aerococcus viridans* |  |  |  | 3 | 3 |
|  | *Anaerococcus* AF408269 96% |  |  |  | 2 | 2 |
|  | *Anaerococcus* AF542229 |  | 1 |  |  | 1 |
|  | *Anaerococcus* AF542229 97% |  |  |  | 1 | 1 |
|  | *Anaerococcus* AY958786 |  |  |  | 1 | 1 |
|  | *Anaerococcus* AY958800 96% |  |  |  | 2 | 2 |
|  | *Anaerococcus* AY958800 97% | 1 |  | 1 |  | 2 |
|  | *Anaerococcus* AY958826 |  | 2 |  | 2 | 4 |
|  | *Anaerococcus* AY958901 |  |  |  | 3 | 3 |
|  | *Anaerococcus* AY959168 | 6 | 3 |  | 6 | 15 |
|  | *Anaerococcus* AY981208 | 1 |  | 1 | 7 | 9 |
|  | *Anaerococcus* DQ130021 92% |  |  | 1 |  | 1 |
|  | *Anaerococcus* DQ130021 | 1 |  | 1 | 1 | 3 |
|  | *Anaerococcus octavius* |  |  | 1 | 1 | 2 |
|  | *Anaerococcus prevotii* | 2 |  |  |  | 2 |
|  | *Anaerococcus* Y07841 95.3% |  | 2 |  |  | 2 |
|  | *Anaerococcus* Y07841 95.7% |  |  | 3 | 3 | 6 |
|  | *Anaerovorax* AJ251215 90% |  |  | 2 | 3 | 5 |
|  | *Brevibacillus* AJ715380 96% |  |  | 2 |  | 2 |
|  | *Carnobacterium* AJ427446 | 10 |  |  |  | 10 |
|  | *Clostridiaceae* AY916293 |  |  |  | 3 | 3 |
|  | *Clostridiaceae* AY976048 |  |  | 3 |  | 3 |
|  | *Clostridiales* AF287775 |  | 2 |  |  | 2 |
|  | *Clostridiales* AF385510 |  |  |  | 1 | 1 |
|  | *Dialister* DQ130020 | 2 |  |  | 1 | 3 |
|  | *Dialister invisus* |  |  |  | 1 | 1 |
|  | *Dorea longicatena* |  |  | 1 | 1 | 2 |
|  | *Enterococcus faecalis* | 2 |  |  |  | 2 |
|  | *Eremococcus coleocola* | 1 | 1 |  |  | 2 |
|  | *Eubacteriaceae* AY821870 96% |  |  |  | 4 | 4 |
|  | *Facklamia hominis* | 5 |  |  |  | 5 |
|  | *Facklamia languida* | 2 |  |  |  | 2 |
|  | *Faecalibacterium* AY976035 |  |  |  | 2 | 2 |
|  | *Filifactor alocis* |  |  |  | 1 | 1 |
|  | *Finegoldia* AB109769 | 16 | 2 |  | 12 | 30 |
|  | *Firmicutes* AF527773 |  |  |  | 1 | 1 |
|  | *Gemella haemolysans* | 1 | 5 | 1 | 20 | 27 |
|  | *Gemella morbillorum* | 1 |  |  | 1 | 2 |
|  | *Gemella sanguinis* | 1 |  |  | 6 | 7 |
|  | *Granulicatella adiacens* |  | 2 |  | 15 | 17 |
|  | *Granulicatella elegans* | 3 |  | 1 |  | 4 |
|  | *Jeotgalicoccus* AY188939 |  |  |  | 3 | 3 |
|  | *Lachnospira* AY977894 |  | 1 |  |  | 1 |
|  | *Lachnospiraceae* AY975275 |  |  |  | 2 | 2 |
|  | *Lachnospiraceae* AY983852 |  |  |  | 1 | 1 |
|  | *Lactobacillus crispatus* | 9 |  |  |  | 9 |
|  | *Lactobacillus delbrueckii subsp. bulgaricus* |  | 1 |  |  | 1 |
|  | *Lactobacillus jensenii* | 3 |  |  |  | 3 |
|  | *Lactobacillus sp.* | 2 |  |  | 2 | 4 |
|  | *Leuconostoc argentinum* | 1 |  |  | 5 | 6 |
|  | *Macrococcus* AY119686 92% |  |  |  | 1 | 1 |
|  | *Papillibacter* U81642 |  |  | 1 |  | 1 |
|  | *Peptoniphilus* AF481225 |  |  |  | 2 | 2 |
|  | *Peptoniphilus* AY958780 96% |  |  |  | 1 | 1 |
|  | *Peptoniphilus harei* | 3 |  | 2 | 9 | 14 |
|  | *Peptoniphilus lacrimalis* |  |  | 1 |  | 1 |
|  | *Peptoniphilus* X90471 88% |  |  |  | 9 | 9 |
|  | *Peptoniphilus* Y07840 93% | 1 |  |  |  | 1 |
|  | *Peptostreptococcus anaerobius* | 1 |  |  |  | 1 |
|  | *Roseburia* AY974841 |  |  |  | 6 | 6 |
|  | *Ruminococcus* AY916259 |  |  |  | 1 | 1 |
|  | *Ruminococcus* AY975494 |  |  |  | 1 | 1 |
|  | *Selenomonas* AY349404 | 4 |  |  |  | 4 |
|  | *Selenomonas sputigena* |  |  |  | 1 | 1 |
|  | *Staphylococcus* AF322002 |  |  | 6 | 3 | 9 |
|  | *Staphylococcus aureus* |  |  | 7 | 37 | 44 |
|  | *Staphylococcus capitis* | 13 | 5 | 70 | 15 | 103 |
|  | *Staphylococcus caprae* | 43 | 17 | 18 | 23 | 101 |
|  | *Staphylococcus cohnii* | 1 |  |  |  | 1 |
|  | *Staphylococcus epidermidis* | 36 | 10 | 20 | 29 | 95 |
|  | *Staphylococcus haemolyticus* | 2 | 29 | 7 | 5 | 43 |
|  | *Staphylococcus hominis* | 20 | 11 | 56 | 120 | 207 |
|  | *Staphylococcus lugdunensis* |  | 2 |  |  | 2 |
|  | *Staphylococcus pasteuri* |  | 2 |  |  | 2 |
|  | *Staphylococcus saccharolyticus* | 7 | 6 |  | 11 | 24 |
|  | *Staphylococcus simulans* |  |  | 6 |  | 6 |
|  | *Staphylococcus warneri* | 14 | 4 | 4 | 4 | 26 |
|  | *Streptococcus* AF429766 |  | 8 |  | 12 | 20 |
|  | *Streptococcus agalactiae* | 1 |  | 1 | 14 | 16 |
|  | *Streptococcus anginosus* |  |  |  | 1 | 1 |
|  | *Streptococcus australis* |  | 1 |  | 6 | 7 |
|  | *Streptococcus cristatus* | 2 |  |  |  | 2 |
|  | *Streptococcus* DQ016719 |  |  |  | 1 | 1 |
|  | *Streptococcus gordonii* | 1 | 3 |  |  | 4 |
|  | *Streptococcus infantis* | 2 | 1 | 1 | 18 | 22 |
|  | *Streptococcus intermedius* | 1 | 4 |  |  | 5 |
|  | *Streptococcus mitis* | 33 | 47 | 7 | 74 | 161 |
|  | *Streptococcus mutans* |  |  | 3 | 1 | 4 |
|  | *Streptococcus oralis* |  |  | 2 | 4 | 6 |
|  | *Streptococcus parasanguinis* | 2 | 5 | 1 | 22 | 30 |
|  | *Streptococcus pseudopneumoniae* |  | 1 | 1 | 1 | 3 |
|  | *Streptococcus salivarius* | 10 | 18 | 2 | 30 | 60 |
|  | *Streptococcus sanguinis* | 19 | 6 | 3 | 3 | 31 |
|  | *Streptococcus* AY959092 |  | 1 |  | 1 | 2 |
|  | *Subdoligranulum* AJ408989 |  |  |  | 1 | 1 |
|  | *Trichococcus pasteurii* |  |  |  | 5 | 5 |
|  | *Veillonella* DQ087189 |  |  |  | 1 | 1 |
|  | *Veillonella* AF186071 91% | 1 |  |  |  | 1 |
|  | *Veillonella atypica* |  |  |  | 2 | 2 |
|  | *Veillonella dispar* | 2 | 3 |  | 3 | 8 |
|  | *Veillonella parvula* | 1 |  |  | 7 | 8 |
|  | **Subtotal** | **290** | **208** | **237** | **605** | **1340** |
| **Fusobacteria**  **(n=4)** | *Fusobacterium* AY278617 |  | 1 |  |  | 1 |
|  | *Fusobacterium* AJ810271 |  |  |  | 1 | 1 |
|  | *Fusobacterium nucleatum* |  |  |  | 1 | 1 |
|  | *Leptotrichia* AF432138 |  |  |  | 1 | 1 |
|  | **Subtotal** |  | **1** |  | **3** | **4** |
| **Planctomycetes**  **(n=1)** | *Blastopirellula* DQ372846 93% |  |  |  | 1 | 1 |
|  | **Subtotal** |  |  |  | **1** | **1** |
| **Proteobacteria**  **(n=111)** | *Achromobacter xylosoxidans* |  |  |  | 2 | 2 |
|  | *Acidovorax temperans* | 1 | 7 | 2 | 12 | 22 |
|  | *Acidovorax* AM084006 | 4 | 3 |  | 1 | 8 |
|  | *Acinetobacter calcoaceticus* |  | 12 |  |  | 12 |
|  | *Acinetobacter* DQ256337 |  |  |  | 2 | 2 |
|  | *Acinetobacter* DQ336974 |  |  |  | 6 | 6 |
|  | *Acinetobacter* DQ337025 |  | 4 |  |  | 4 |
|  | *Acinetobacter haemolyticus* | 9 |  |  |  | 9 |
|  | *Acinetobacter johnsonii* | 1 | 2 |  |  | 3 |
|  | *Acinetobacter junii* | 30 | 4 | 5 | 10 | 49 |
|  | *Acinetobacter parvus* |  | 1 |  | 2 | 3 |
|  | *Acinetobacter ursingii* | 5 | 8 |  | 1 | 14 |
|  | *Acinetobacter* Z93987 |  |  | 1 |  | 1 |
|  | *Alishewanella* AF371859 |  |  |  | 1 | 1 |
|  | *Alishewanella fetalis* |  |  | 1 |  | 1 |
|  | *Alkanindiges* AF513979 93% | 5 |  |  |  | 5 |
|  | *Alkanindiges* AY251390 94% |  | 3 |  |  | 3 |
|  | *Amaricoccus* Y09610 94% | 1 |  |  |  | 1 |
|  | *Aquabacterium* AB128892 | 2 |  |  |  | 2 |
|  | *Aquabacterium* AY212659 |  | 2 |  | 4 | 6 |
|  | *Bacteroides ureolyticus* |  |  |  | 1 | 1 |
|  | *Bdellovibrio* AF148938 88% | 4 |  |  |  | 4 |
|  | *Bdellovibrio* DQ413094 90% |  | 1 |  |  | 1 |
|  | *Betaproteobacteria* AY360547 | 3 |  |  |  | 3 |
|  | *Bosea minatitlanensis* |  |  |  | 1 | 1 |
|  | *Bradyrhizobiaceae* U87763 | 2 |  |  |  | 2 |
|  | *Brevundimonas aurantiaca* | 2 |  |  |  | 2 |
|  | *Brevundimonas vesicularis* | 1 | 2 |  |  | 3 |
|  | *Burkholderia cenocepacia* |  |  |  | 1 | 1 |
|  | *Burkholderiales* DQ016727 | 4 |  |  |  | 4 |
|  | *Burkholderiales* AJ412678 96% | 4 |  |  |  | 4 |
|  | *Burkholderiales* AY005030 |  |  |  | 1 | 1 |
|  | *Caulobacteraceae* AJ459874 | 1 |  |  |  | 1 |
|  | *Citrobacter farmeri* |  |  | 3 | 2 | 5 |
|  | *Comamonas denitrificans* |  | 1 | 7 | 8 | 16 |
|  | *Comamonas kerstersii* |  |  |  | 2 | 2 |
|  | *Delftia acidovorans* |  |  | 9 | 6 | 15 |
|  | *Devosia* AJ863377 |  | 1 |  |  | 1 |
|  | *Diaphorobacter nitroreducens* | 7 | 8 | 9 | 12 | 36 |
|  | *Enhydrobacter aerosaccus* | 34 | 102 | 1 | 10 | 147 |
|  | *Enterobacter aerogenes* |  | 1 |  |  | 1 |
|  | *Enterobacter asburiae* | 1 |  |  |  | 1 |
|  | *Gammaproteobacteria* AY922146 | 1 |  |  |  | 1 |
|  | *Haemophilus* AY005033 |  | 2 |  |  | 2 |
|  | *Haemophilus* AY975706 | 5 | 7 | 2 | 11 | 25 |
|  | *Haemophilus* AF224309 | 2 |  |  |  | 2 |
|  | *Haemophilus aphrophilus* |  |  |  | 1 | 1 |
|  | *Haemophilus influenzae* |  |  |  | 10 | 10 |
|  | *Hydrogenophilus thermoluteolus* |  |  | 1 |  | 1 |
|  | *Hyphomicrobium facile* | 1 |  |  |  | 1 |
|  | *Incertae sedis* DQ297980 |  | 1 |  |  | 1 |
|  | *Janthinobacterium lividum* |  | 1 |  |  | 1 |
|  | *Lysobacter* AY074793 94% |  | 1 |  |  | 1 |
|  | *Mesorhizobium* AP003001 95% | 1 |  |  |  | 1 |
|  | *Methylobacillus* AF289159 94% | 7 |  |  |  | 7 |
|  | *Methylobacterium* AY592150 | 1 |  |  |  | 1 |
|  | *Methylobacterium* AY741717 | 1 |  |  |  | 1 |
|  | *Methylobacterium extorquens* | 1 |  |  |  | 1 |
|  | *Methylobacterium mesophilicum* | 1 |  |  |  | 1 |
|  | *Methylobacterium* Z23159 |  | 1 |  |  | 1 |
|  | *Neisseria* AJ239301 |  | 1 |  | 1 | 2 |
|  | *Neisseria* AJ786809 |  |  | 2 | 5 | 7 |
|  | *Neisseria* DQ409137 | 1 |  |  | 1 | 2 |
|  | *Neisseriaceae* AY225604 91% |  | 1 |  |  | 1 |
|  | *Neisseriaceae* AY225604 | 18 | 1 |  |  | 19 |
|  | *Novosphingobium subterraneum* |  | 1 |  |  | 1 |
|  | *Paracoccus* AJ619068 | 1 |  |  |  | 1 |
|  | *Paracoccus aminovorans* |  | 1 |  |  | 1 |
|  | *Paracraurococcus*  AF443585 93% | 1 |  |  |  | 1 |
|  | *Paracraurococcus* AF443585 95% | 2 |  |  |  | 2 |
|  | *Pasteurellaceae* AJ290758 | 1 |  |  |  | 1 |
|  | *Pasteurellaceae* AY005034 | 1 |  |  |  | 1 |
|  | *Pedomicrobium australicum* | 4 |  |  |  | 4 |
|  | *Pseudomonas aeruginosa* | 5 |  | 1 | 1 | 7 |
|  | *Pseudomonas* AF326380 | 1 |  |  |  | 1 |
|  | *Pseudomonas* AJ312163 |  |  |  | 1 | 1 |
|  | *Pseudomonas* AJ575816 | 3 |  |  |  | 3 |
|  | *Pseudomonas azotoformans* |  |  |  | 2 | 2 |
|  | *Pseudomonas balearica* |  |  |  | 1 | 1 |
|  | *Pseudomonas costantinii* |  | 1 |  | 2 | 3 |
|  | *Pseudomonas* DQ130043 | 4 |  |  | 1 | 5 |
|  | *Pseudomonas koreensis* |  |  |  | 1 | 1 |
|  | *Pseudomonas monteilii* |  | 1 | 4 | 3 | 8 |
|  | *Pseudomonas plecoglossicida* |  |  | 2 | 2 | 4 |
|  | *Pseudomonas pseudoalcaligenes* |  |  |  | 1 | 1 |
|  | *Pseudomonas putida* |  |  | 1 | 1 | 2 |
|  | *Pseudomonas saccharophila* | 12 | 8 |  |  | 20 |
|  | *Pseudomonas stutzeri* | 21 | 6 | 7 | 2 | 36 |
|  | *Pseudomonas tremae* | 3 |  |  |  | 3 |
|  | *Ralstonia insidiosa* |  | 1 |  |  | 1 |
|  | *Ralstonia pickettii* |  |  |  | 4 | 4 |
|  | *Rhizobiales* AB121772 96% | 2 |  |  |  | 2 |
|  | *Rhizobiales* AF358012 | 1 |  |  |  | 1 |
|  | *Rhizobium giardinii* |  | 2 |  |  | 2 |
|  | *Rhodocyclaceae* AY953150 |  |  |  | 2 | 2 |
|  | *Serratia liquefaciens* | 4 |  |  |  | 4 |
|  | *Serratia marcescens subsp. sakuensis* | 2 |  |  |  | 2 |
|  | *Shigella dysenteriae* |  | 1 | 1 |  | 2 |
|  | *Shigella flexneri* |  |  | 2 | 7 | 9 |
|  | *Sphingobium amiense* | 2 |  |  |  | 2 |
|  | *Sphingomonas* AJ555475 |  | 1 |  |  | 1 |
|  | *Sphingomonas anadarae* |  |  |  | 3 | 3 |
|  | *Sphingomonas oligophenolica* |  | 2 |  |  | 2 |
|  | *Sphingomonas yabuuchiae* |  | 1 |  |  | 1 |
|  | *Sphingomonas taejonensis* |  | 2 |  |  | 2 |
|  | *Sphingopyxis* AY328823 | 2 | 1 |  |  | 3 |
|  | *Stenotrophomonas acidaminiphila* |  |  |  | 1 | 1 |
|  | *Stenotrophomonas maltophilia* | 1 |  |  | 2 | 3 |
|  | *Wautersia metallidurans* |  | 2 | 1 |  | 3 |
|  | *Xanthomonadaceae* AJ619045 94% | 3 |  |  |  | 3 |
|  | *Xanthomonadaceae* AB008508 |  | 1 |  |  | 1 |
|  | **Subtotal** | **237** | **210** | **62** | **151** | **660** |
| **Thermomicrobia**  **(n=1)** | *Thermomicrobium* AY250886 93% | 1 |  |  |  | 1 |
|  | **Subtotal** | **1** |  |  |  | **1** |
| **TM7**  **(n=6)** | *TM7* AY349415 |  | 1 |  |  | 1 |
|  | *TM7* AF385520 |  |  |  | 1 | 1 |
|  | *TM7* AY005446 |  |  |  | 2 | 2 |
|  | *TM7* DQ248299 95% |  | 2 |  |  | 2 |
|  | *TM7* AF385500 |  | 2 |  |  | 2 |
|  | *TM7* AB195911 94% |  | 1 |  |  | 1 |
|  | **Subtotal** |  | **6** |  | **3** | **9** |
| **Unclassified**  **(n=2)** | Bacteria AJ619064 87% | 1 |  |  |  | 1 |
|  | Bacteria AJ619064 | 8 | 1 |  |  | 9 |
|  | **Subtotal** | **9** | **1** |  |  | **10** |
| **Total** | | **1,221** | **817** | **611** | **1,314** | **3,963** |
| **Number of SLOTU** | | **182** | **130** | **95** | **188** | **366** |

^a^NNT1: 12 samples from six healthy people, reported in a prior study (11).

^b^NNT2: An additional eight samples from four of the six healthy people were collected 8-

10 months later.

^c^PN: Six samples from normal skin from the six patients with psoriasis.

^d^PP: 13 samples from psoriatic lesions from the six patients with psoriasis.
