# Supplementary material for: Substantial Alterations of the Cutaneous Bacterial Biota in Psoriatic Lesions
Source: PLoS One. 2008 Jul 23;3(7):e2719. doi: 10.1371/journal.pone.0002719 (PMC2447873; doi:10.1371/journal.pone.0002719)
Supplement: Table S3 — The four most common species found in different groups of skin specimens (0.04 MB DOC) [file pone.0002719.s003.doc]

**Table S3. The four most common species found in different groups of skin specimens**

|  | **Percent of total clones (rank)** ^e^ | | | |
| --- | --- | --- | --- | --- |
| **Species** | **NNT1**^a^ | **NNT2**^b^ | **PN**^c^ | **PP**^d^ |
| *Propionibacterium acnes* | **20.6(1)** | **19.5(1)** | **11.8(1)** | 2.5(7) |
| *Corynebacterium tuberculostearicum* | **6.4(2)** | 3.3(6) | **11.6(2)** | **11.4(1)** |
| *Staphylococcus hominis* | 1.6(12) | 1.3(11) | **9.2(4)** | **9.1(2)** |
| *Streptococcus mitis* | 2.7(6) | **5.8(3)** | 1.1(15) | **5.6(3)** |
| *Enhydrobacter aerosaccus* | 2.8(5) | **12.5(2)** | 0.2(58) | 0.8(32) |
| *Staphylococcus capitis* | 1.1(20) | 0.6(29) | **11.5(3)** | 1.1(19) |
| *Staphylococcus caprae* | **3.5(3)** | 2.1(9) | 2.9(7) | 1.8(11) |
| *Staphylococcus epidermidis* | **2.9(4)** | 1.2(12) | 3.3(6) | 2.2(9) |
| *Corynebacterium simulans* | 0.7(31) | 0.2(51) | 4.4(5) | **3.4(4)** |
| *Dermacoccus* AF409025 | 0.1(116) | **5.8(3)** | 0 | 1.4(17) |
| ***Four most common species*** | ***33.5*** | ***43.5*** | ***44.0*** | ***29.6*** |

^a^NNT1: 12 samples from six healthy people, reported in a prior study (11).

^b^NNT2: Eight samples from four of six healthy people 8-10 months later.

^c^PN: Six samples from normal skin from the six patients with psoriasis.

^d^PP: 13 samples from psoriatic lesions from the six patients with psoriasis.

^e^**Bold** indicates that species is one of the four most common for that group of samples;

number in parentheses indicates rank order in that group of samples.
