# Supplementary material for: Substantial Alterations of the Cutaneous Bacterial Biota in Psoriatic Lesions
Source: PLoS One. 2008 Jul 23;3(7):e2719. doi: 10.1371/journal.pone.0002719 (PMC2447873; doi:10.1371/journal.pone.0002719)
Supplement: Table S4 — Supplemental information (0.03 MB DOC) [file pone.0002719.s004.doc]

**Table S4. Distribution of gram-positive and gram-negative anaerobic and facultative bacterial species in six patients with psoriasis, in relation to skin lesion status**

| **Classification ^a^** | **Percent of clones** | | **P-value** |
| --- | --- | --- | --- |
|  | **(PN)**  **Normal skin (n=611) ^b^** | **(PP)**  **Psoriatic lesions**  **(n=1,314)^b^** |  |
| G^+^ facultative aerobe | 70.5 | 72.1 | 0.98 |
| G^-^ facultative aerobe | 12.9 | 14.2 | 0.97 |
| G^+^ anaerobe | 16.2 | 10.2 | 0.003 |
| *Propionibacterium species* | 13.1 | 2.8 | <0.001 |
| Other | 3.1 | 7.4 | 0.13 |
| G^-^ anaerobe | 0.3 | 3.6 | 0.02 |

^a^The status of uncharacterized SLOTUs (n=14) was inferred from that of their closest neighbor.

^b^ Number of clones studied.
