# Supplementary material for: Substantial Alterations of the Cutaneous Bacterial Biota in Psoriatic Lesions
Source: PLoS One. 2008 Jul 23;3(7):e2719. doi: 10.1371/journal.pone.0002719 (PMC2447873; doi:10.1371/journal.pone.0002719)
Supplement: Table S5 — Origin of skin samples (0.10 MB DOC) [file pone.0002719.s005.doc]

**Table S5. Origin of skin samples**

| **Subjects** | | | | | | **Number of clones** | |  | | | |
| --- | --- | --- | --- | --- | --- | --- | --- | --- | --- | --- | --- |
| **Code** | **Age**  **(years)** | **Gender** | **Skin sample** | **Source of**  **sample** | **Sequences**  **obtained ^a^** | | **Included in**  **analysis ^b^** |  | | | |
| A | 41 | Female | Normal | Forearm, Left | 120 | | 105 |  | | | |
|  |  |  |  | Right | 119 | | 103 |  | | | |
| B | 21 | Female | Normal | Left | 148 | | 103 |  | | | |
|  |  |  |  | Right | 150 | | 101 |  | | | |
| C | 31 | Female | Normal | Left | 121 | | 103 |  | | | |
|  |  |  |  | Right | 104 | | 99 |  | | | |
| D | 53 | Male | Normal | Left | 112 | | 101 |  | | | |
|  |  |  |  | Right | 114 | | 103 |  | | | |
| E | 54 | Male | Normal | Left | 105 | | 100 |  | | | |
|  |  |  |  | Right | 104 | | 103 |  | | | |
| F | 29 | Male | Normal | Left | 113 | | 102 |  | | | |
|  |  |  |  | Right | 106 | | 98 |  | | | |
| A2 | 41 | Female | Normal | Left | 106 | | 100 |  | | | |
|  |  |  |  | Right | 108 | | 103 |  | | | |
| C2 | 31 | Female | Normal | Left | 106 | | 101 |  | | | |
|  |  |  |  | Right | 106 | | 105 |  | | | |
| E2 | 54 | Male | Normal | Left | 104 | | 103 | Duration of psoriasis (years) | Body surface area involved (%) | Family history | Psoriatic arthritis |
|  |  |  |  | Right | 106 | | 99 |  |  |  |  |
| F2 | 29 | Male | Normal | Left | 106 | | 102 |  |  |  |  |
|  |  |  |  | Right | 106 | | 104 |  |  |  |  |
| 1P | 55 | Male | Psoriasis | Finger, Right | 103 | | 103 | 24 | 5 | + | - |
|  |  |  |  | Elbow, Right | 107 | | 104 |  |  |  |  |
|  |  |  | Normal | Forearm, Right | 106 | | 102 |  |  |  |  |
| 2P | 52 | Male | Psoriasis | Arm, Left | 104 | | 100 | NA^c^ | NA | NA | NA |
|  |  |  |  | Leg, Left | 106 | | 97 |  |  |  |  |
|  |  |  |  | Forearm, Left | 107 | | 101 |  |  |  |  |
|  |  |  | Normal | Shoulder, Left | 106 | | 101 |  |  |  |  |
| 3P | 25 | Male | Psoriasis | Leg, Left | 107 | | 102 | 1 | 15 | - | + |
|  |  |  |  | Arm, Left | 109 | | 98 |  |  |  |  |
|  |  |  | Normal | Forearm, Right | 105 | | 103 |  |  |  |  |
| 4P | 48 | Female | Psoriasis | Elbow, Left | 109 | | 100 | 20 | 10 | + | - |
|  |  |  |  | Forearm, Left | 120 | | 104 |  |  |  |  |
|  |  |  | Normal | Knee, Left | 104 | | 102 |  |  |  |  |
| 6P | 31 | Female | Psoriasis | Forearm, Right | 103 | | 102 | 5 | 10 | - | + |
|  |  |  |  | Back | 103 | | 101 |  |  |  |  |
|  |  |  | Normal | Forearm, Right | 103 | | 103 |  |  |  |  |
| 8P | 67 | Female | Psoriasis | Arm, Right | 103 | | 101 | 7 | 20 | - | + |
|  |  |  |  | Abdomen | 105 | | 100 |  |  |  |  |
|  |  |  | Normal | Forearm, Right | 104 | | 101 |  |  |  |  |
| **Total** | | | | | | **4,278** | **3,963** |  |  |  |  |

^a^ Includes contaminants, chimerae, and sequences of low quality that were excluded.

^b^ Specimens from subjects A-F were reported in a prior study (11)

^c^ Not available
