# Supplementary figures and images for: Substantial Alterations of the Cutaneous Bacterial Biota in Psoriatic Lesions
Source: PLoS One. 2008 Jul 23;3(7):e2719. doi: 10.1371/journal.pone.0002719 (PMC2447873; doi:10.1371/journal.pone.0002719)

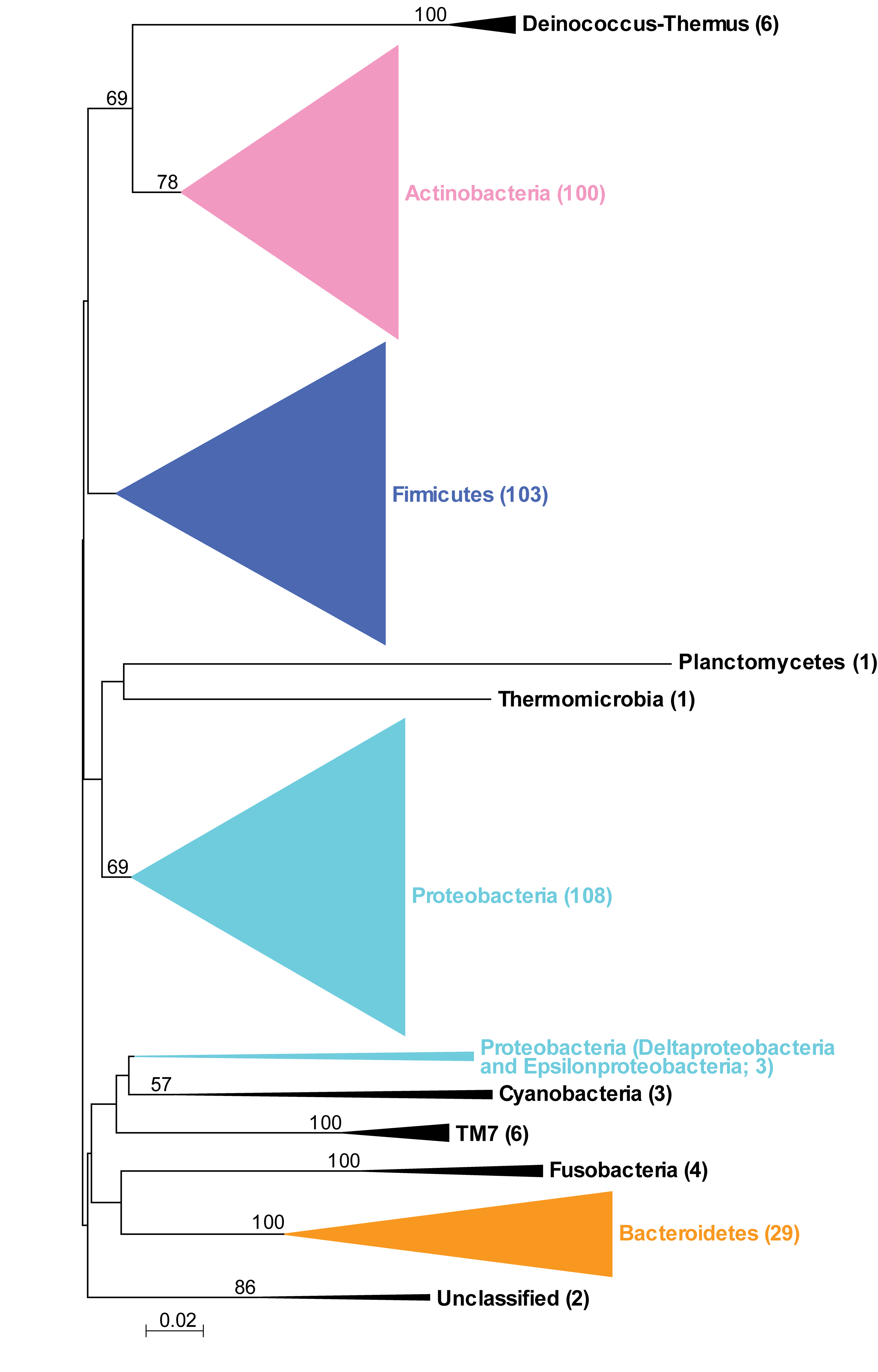

Supplement: Figure S1 — Phylogenetic analysis of bacterial 16S rDNA detected in 39 samples from human skin of 12 persons. From 3,963 clones, sequences representing 11 bacterial phyla and 366 SLOTUs were observed. The numbers in parentheses indicate the number of SLOTUs in each phylum. Alignments were done with Greengenes, and misalignments were manually in MEGA 4.0 (38), evolutionary distances were calculated with the Jukes-Cantor algorithm, and phylogenetic trees were determined by the Neighbor-Joining method; with 1,000 trees generated, bootstrap confidence levels are shown at tree nodes for values ≥50%. (0.78 MB TIF) [file pone.0002719.s006.tif]

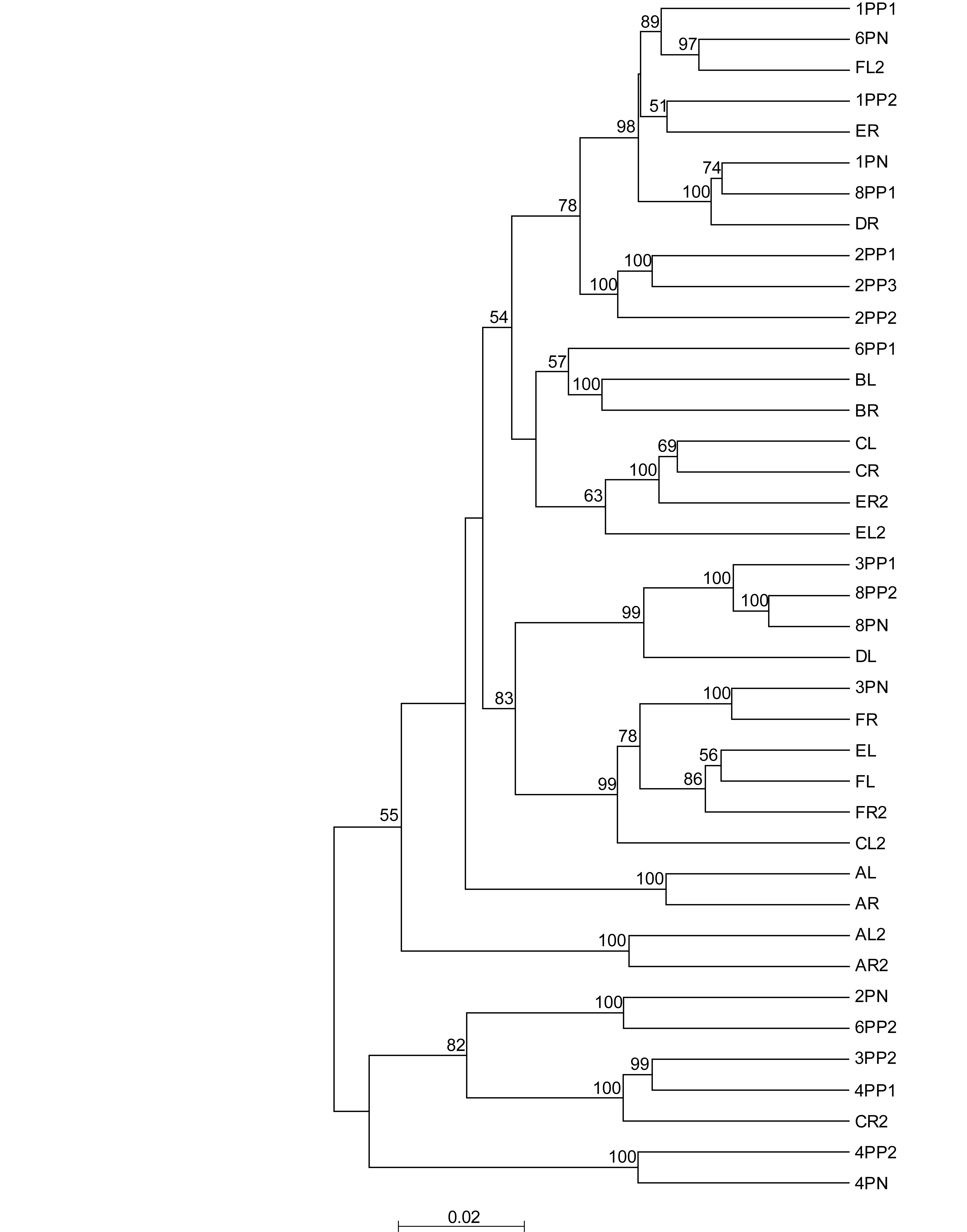

Supplement: Figure S2 — Hierarchical clustering of 39 human skin samples from healthy and psoriasis subjects using weighted Unifrac. The numerical support for nodes present in ≥50% of sequence jackknifing is indicated, based on 1,000 permutations. The healthy subjects were designated A–F, and at each sampling both left (L) and right (R) forearm skin was examined. In four subjects, new specimens were obtained 8–10 months later (e.g. designated AL2). The psoriatic patients were designated 1, 2, 3, 4, 6, and 8, PN indicates that sample is from uninvolved skin and PP is from psoriatic lesions. The highly significant clustering observed relates to samples from the same person. (1.11 MB TIF) [file pone.0002719.s007.tif]
